# Transcriptome analysis of genes involved in anthocyanins biosynthesis and transport in berries of black and white spine grapes (*Vitis davidii*)

**DOI:** 10.1186/s41065-016-0021-1

**Published:** 2016-12-07

**Authors:** Lei Sun, Xiucai Fan, Ying Zhang, Jianfu Jiang, Haisheng Sun, Chonghuai Liu

**Affiliations:** Zhengzhou Fruit Research Institute, Chinese Academy of Agricultural Sciences, Zhengzhou, 450009 China

**Keywords:** *Vitis davidii*, Berry skin, Anthocyanins, Transcriptome analysis, Candidate genes

## Abstract

**Background:**

The color of berry skin is an important economic trait for grape and is essentially determined by the components and content of anthocyanins. The fruit color of Chinese wild grapes is generally black, and the profile of anthocyanins in Chinese wild grapes is significantly different from that of *Vitis vinifera*. However, *V. davidii* is the only species that possesses white berry varieties among Chinese wild grape species. Thus, we performed a transcriptomic analysis to compare the difference of transcriptional level in black and white *V. davidii*, in order to find some key genes that are related to anthocyanins accumulation in *V. davidii*.

**Results:**

The results of anthocyanins detection revealed that 3,5-*O*-diglucoside anthocyanins is the predominant anthocyanins in *V. davidii*. It showed obvious differences from *V. vinifera* in the profile of the composition of anthocyanins. The transcriptome sequencing by Illumina mRNA-Seq technology generated an average of 57 million 100-base pair clean reads from each sample. Differential gene expression analysis revealed thousands of differential expression genes (DEGs) in the pairwise comparison of different fruit developmental stages between and within black and white *V. davidii*. After the analysis of functional category enrichment and differential expression patterns of DEGs, 46 genes were selected as the candidate genes. Some genes have been reported as being related to anthocyanins accumulation, and some genes were newly found in our study as probably being related to anthocyanins accumulation. We inferred that *3AT* (VIT_03s0017g00870) played an important role in anthocyanin acylation, *GST4* (VIT_04s0079g00690) and *AM2* (VIT_16s0050g00910) played important roles in anthocyanins transport in *V. davidii*. The expression of some selected DEGs was further confirmed by quantitative real-time PCR (qRT-PCR).

**Conclusions:**

The present study investigated the transcriptomic profiles of berry skin from black and white spine grapes at three fruit developmental stages by Illumina mRNA-Seq technology. It revealed the variety specificity of anthocyanins accumulation in *V. davidi* at the transcriptional level. The data reported here will provide a valuable resource for understanding anthocyanins accumulation in grapes, especially in *V. davidii*.

**Electronic supplementary material:**

The online version of this article (doi:10.1186/s41065-016-0021-1) contains supplementary material, which is available to authorized users.

## Background

Grapevine (*Vitis* L.) is an important economic crop used as table fruit, dried raisins, and for wines or juice. The color of berry skin is an important economic trait for grapes. And it is closely related to the components and content of anthocyanins [1, 2]. In recent years, the profile of anthocyanins in grape berry skin has been widely studied. The anthocyanins are glycosides and acylglycosides of anthocyanidins. The main anthocyanidins found in grapes are pelargonidin, cyanidin, delphinidin, peonidin, petunidin as well as malvidin which is usually the predominant anthocyanidin in most red grapes [3, 4]. However, the kind of anthocyanins exhibits obvious differences in different grape species [5]. In the *V. vinifera* cultivars, only 3-*O*-monoglucoside anthocyanins are detected, and just a few cultivars produce pelargonidin-based anthocyanins [6, 7]. In non-*V. vinifera* species, 3,5-*O*-disglucoside anthocyanins widely exist, even the pelargonidin-based anthocyanins are also detected [7, 8]. In addition, most *V. vinifera* cultivars possess acylated forms of the anthocyanins, but some other grape species do not produce acylated forms of anthocyanins, such as *V. rotundifolia* [9].

Anthocyanins in grape berry skin are synthesized via the flavonoid pathways, which has been extensively studied. In fact, the synthesis of anthocyanins shares the same upstream pathways with proanthocyanidins and flavonol derivatives [10]. Phenylalanine is the precursor of flavonoid, which is used as substrate, phenylalanine ammonia lyase (PAL), cinnamate-4-hydroxylase (C4H) and 4-coumaroyl-CoA synthase (4CL) catalyze a series of reactions to produce 4-coumaroyl-CoA. The catalysis of chalcone synthase (CHS) is the first committed step in the flavonoid biosynthetic pathway, which can catalyze the synthesis of chalcones [10]. Subsequently, after the action of chalcone isomerase (CHI), the basic three rings of the general C6-C3-C6 flavonoid skeleton is produced [11]. The B ring of the naringenin flavanone can be further hydroxylated by flavonoid -3′-hydroxylase(F3′H) or flavonoid-3′5′-hydroxylase (F3′5′H) to form eriodictyol or pentahydroxyflavanone [12, 13]. The naringenin, eriodictyol and pentahydroxyflavanone can be modified by the catalysis of flavanone-3β-hydroxylase (F3H) to form the corresponding dihydrokaempferol, dihydroquercetin and dihydromyricetin, respectively. Besides, the dihydrokaempferol can also be catalyzed by F3′H or F3′5′H to produce other two dihydroflavonols, dihydroquercetin or dihydromyricetin [1]. Then, dihydroflavonol-4-reductase (DFR) catalyzes these dihydroflavonols to form their corresponding leucoanthocyanidins [1]. In the past, leucoanthocyanidin dioxygenase (LDOX) used to be considered as the first key enzyme that could catalyze the formation of anthocyanidins [14, 15] and lead the flavonoid flux into the anthocyanin branch. However, more studies showed that LDOX also play an important role in the biosynthesis of proanthocyanidins [16–19]. And the anthocyanidins which are the production of LDOX can also be catalyzed by anthocyanidin reductase (ANR) to produce the substrates for the proanthocyanidins synthesis [20, 21]. The glycosylation, methylation and acylation of anthocyanidins are very important for their stabilization. Glycosylation of the anthocyanidins is the key step to produce anthocyanins which is catalyzed by the UDP-glucose: anthocyanidin: flavonoid glucosyltransferase (UFGT) [1, 22–25]. In *V. vinifera*, the anthocyanidins can only be catalyzed by UFGT to glycosylate at C3 position [24]. Thus, it is also called 3GT. The S-adenosyl-L-methionine (SAM) or *O*-methyltransferase (OMT) can catalyze the methylation of the hydroxyl groups at the C3 positions or both at the C3 and C5 positions on the B rings of the anthocyanins [26, 27]. Acylation can greatly enhance the structural diversity and stability of anthocyanins [28]. It is catalyzed by the action of anthocyanin acyltransferases (AAT) [3, 29]. There are mainly two types of ACTs that are classified based on the acyl group donors: the BAHD family using acyl-CoA and the serine carboxypeptidase-like(SCPL) group using acyl-activated sugars [30, 31]. Anthocyanins are synthesized in the cytoplasm but accumulate in the vacuoles. Recent researches showed that glutathione S-transferase (GST), multidrug resistance-associated protein (MRP) and multidrug and toxic compound extrusion (MATE) were closely related to the transport of anthocyanins [32].

As part of the flavonoid pathway, the synthesis of anthocyanins are regulated by a complex regulation at the transcriptional level [4, 10, 15, 33–35]. Generally, the flavonoid pathway of anthocyanins biosynthesis is under the control of Myb transcriptional factors, basic helix–loop-helix proteins(bHLH) and WD40-like proteins, which also play crucial roles in the regulation of flavonols and proanthocyanidins [10, 17–19, 36–38]. The research showed that the *bHLH* gene, *VvMYC1*, was characterized as a component of the transcriptional complex regulating anthocyanins biosynthesis in grapevine [39]. In grapes, a series of R2R3-Myb transcriptional factors are related to the synthesis of anthocyanins [40]. The first Myb transcriptional factor Myba in grape was identified and isolated from *V. labrusca* hybrids [41], and the results suggested that VlmybA1-1, VlmybA1-2 and VlmybA2 transcriptional factors are involved in the regulation of anthocyanin biosynthesis in the grape by regulating the expression of the *UFGT* gene [41–43]. In red *V. vinifera* grapes, the functional VvmybA1 can regulate the expression of the *UFGT* gene to promote the synthesis of anthocyanin. However, in white *V. vinifera* grapes, a retrotransposon (*Gret1*) is inserted in the 5′-flanking region of *VvmybA1* gene to form a non-functional *VvmybA1a* gene, resulting in the transcription factor losing its function [44]. Subsequently, two other *VvmybA* regulator genes, *VvmybA2* and *VvmybA3* have been cloned and identified [45]. The *VvmybA1* and *VvmybA2* are very similar, both of them can regulate the accumulation of anthocyanins in the grape berries, and the white-fruited grapes are caused by the mutation of these two similar and adjacent regulatory genes [46, 47]. More recent researches suggest that variation in anthocyanins content in grapes is involved with the *VvmybA* gene cluster [48–50].

China is the concentrated distribution area of East Asian *Vitis* species, which has more than 35 *Vitis* species [51–53]. In recent years, the research of anthocyanins in Chinese wild grapes has been carried out, and the profile of anthocyanins in Chinese wild grapes is significantly different from *V. vinifera* [5, 7, 54–56]. Generally, only 3-*O*-monoglucoside anthocyanins are detected in *V. vinifera* cultivars, and almost all of the cultivars of *V. vinifera* are devoid of pelargonidin-based anthocyanins. However, in Chinese wild grapes (such as *V. amurensis*, *V. davidii* and *V. quinquangularis*), the 3,5-*O*-disglucoside anthocyanins widely exist, and pelargonidin-based anthocyanins are detected in some wild species [5, 7, 55, 56]. The berry color of most Chinese wild grapes is black, except for the white-fruited varieties of *V. davidii*. The subsequent research found that *VvmybA1a* gene was detected in white-fruited varieties of *V. davidii* [57]. The white fruit may be caused by this mutation of *mybA1* gene. In order to find the causes that lead to the different profiles of anthocyanins between Chinese wild grapes and *V. vinifera*, we used the black and white varieties of *V. davidii* as the materials, and the method of transcriptome analysis to detect the differences at the transcriptional level that might be involved in anthocyanins accumulation of these two varieties.

## Results

### Anthocyanin composition and content

A total of 24 kinds of anthocyanins were detected at three fruit developmental stages of black and white spine grapes (Table 1). Five categories of anthocyanins were detected: 11 kinds of anthocyanidin diglucosides and 13 kinds of anthocyanidin monoglucosides. And 14 kinds of coumaroylated anthocyanins were detected. The highest content of anthocyanins was detected in the third stage black spine grape (B3, DAF120), it was 1382.127 mg.kg^−1^. In black spine grape, the major anthocyanin was Malvidin-3,5-*O*-diglucoside, which accounted for 87% of the total anthocyanins. Besides, we also detected trace amount of anthocyanins in white *V. davidii*.

**Table 1:** Anthocyanins concentration in three stages of black and white spine grapes

Compound	Anyhocyanins content/(mg.kg^−1^) FW of berry skin					
	B1	B2	B3	W1	W2	W3
Dp-3-glu	0.03 ± 0.001d	0.317 ± 0.006a	0.093 ± 0.006b	0.019 ± 0.002e	0.01 ± 0.001e	0.069 ± 0.003c
Dp-3,5-diglu	nd	2.71 ± 0.243b	10.813 ± 0.307a	nd	0.02 ± 0.003d	1.73 ± 0.098c
Cy-3,5-diglu	0.02 ± 0.003d	0.347 ± 0.065a	0.25 ± 0.04b	nd	0.011 ± 0.001d	0.133 ± 0.015c
Cy-3-glu	0.66 ± 0.046b	0.437 ± 0.067c	1.417 ± 0.095a	0.074 ± 0.009e	0.217 ± 0.031d	0.133 ± 0.012de
Cy-3,5-coum-diglu(trans)	nd	nd	0.039 ± 0.002a	nd	nd	nd
Cy-3-coum-glu(cis)	0.031 ± 0.003b	nd	0.056 ± 0.005a	nd	nd	nd
Cy-3-coum-glu(trans)	0.117 ± 0.006b	nd	0.167 ± 0.015a	nd	nd	nd
Pt-3-glu	0.079 ± 0.005b	0.347 ± 0.045a	0.39 ± 0.046a	0.04 ± 0.003b	0.020 ± 0.004b	0.036 ± 0.004b
Pt-3,5-diglu	nd	0.717 ± 0.050b	7.16 ± 0.120a	nd	nd	0.383 ± 0.051c
Pt-3,5-coum-diglu(trans)	nd	0.079 ± 0.006b	4.203 ± 0.307a	nd	nd	nd
Pt-3-coum-glu(cis)	nd	nd	nd	0.009 ± 0.002a	nd	nd
Pt-3-coum-glu(trans)	0.13 ± 0.01c	0.387 ± 0.021b	3.02 ± 0.193a	0.030 ± 0.003c	0.025 ± 0.006c	0.052 ± 0.009c
Pn-3,5-diglu	0.063 ± 0.006c	9.263 ± 0.091b	26.043 ± 0.419a	0.03 ± 0.006c	0.04 ± 0.002c	0.133 ± 0.006c
Pn-3-glu	2.093 ± 0.104a	0.663 ± 0.0153c	1.263 ± 0.025b	0.05 ± 0d	0.097 ± 0.006d	0.1 ± 0d
Pn-3,5-coum-diglu(cis)	0.01 ± 0.004c	0.1 ± 0.005b	0.763 ± 0.025a	nd	nd	nd
Pn-3,5-coum-diglu(trans)	0.03 ± 0.005c	2.777 ± 0.119b	16.24 ± 0.586a	nd	nd	nd
Pn-3-coum-glu(cis)	0.256 ± 0.009b	0.247 ± 0.012b	0.316 ± 0.006a	0.068 ± 0.002e	0.104 ± 0.001d	0.181 ± 0.007c
Pn-3-coum-glu(trans)	0.817 ± 0.031b	0.237 ± 0.006c	0.88 ± 0.01a	0.08 ± 0.004d	0.04 ± 0.001e	0.05 ± 0.002de
Mv-3,5-diglu	0.113 ± 0.006c	31.827 ± 1.462b	1204.697 ± 5.548a	0.03 ± 0.002c	0.177 ± 0.006c	1.043 ± 0.021c
Mv-3-glu	0.46 ± 0.009c	1.17 ± 0.026b	6.443 ± 0.170a	0.02 ± 0.003d	nd	0.06 ± 0.003d
Mv-3,5-coum-diglu(trans)	nd	nd	0.873 ± 0.021a	nd	nd	nd
Mv-3,5-coum-diglu(trans)	nd	0.24 ± 0.01b	19.743 ± 0.396a	nd	nd	nd
Mv-3-coum-glu(cis)	0.03 ± 0.002b	0.03 ± 0.004b	1.443 ± 0.0351a	nd	nd	nd
Mv-3-coum-glu(trans)	0.147 ± 0.006b	0.787 ± 0.025b	75.8 ± 1.899a	0.03 ± 0.002b	0.02 ± 0.001b	0.03 ± 0.001b
Total	5.096 ± 0.127c	52.690 ± 1.867b	1382.127 ± 4.633a	0.490 ± 0.018c	0.790 ± 0.029c	4.135 ± 0.064c

B1, B2 and B3 refer to the berries of black spine grape of 40DAF, 80DAF and 120DAF, respectively. W1, W2 and W3 refer to the berries of white spine grape of 40DAF, 80DAF and 120DAF, respectively

*Dp* delphinidin, *Cy* cyanidin, *Pt* petunidin, *Pn* peonidin, *Mv* malvidin, *glu* glucoside, *diglu* diglucoside, *coum* coumaroyl, *nd* not detected; A significance level of *p* < 0.01 was applied

### mRNA sequencing

**Fig. 1:**
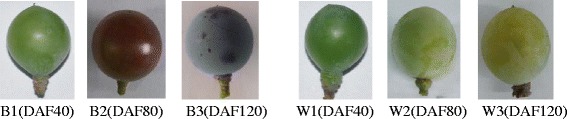
Berries of black and white spine grapes at different ripening stages. B1, B2 and B3 refer to the berries of black spine grape of 40 DAF, 80 DAF and 120 DAF. W1, W2 andW3 refer to the berries of white spine grape of 40 DAF, 80 DAF and 120 DAF

A total of 12 cDNA libraries were constructed from the total RNA of black and white spine grape berry skin in three fruit developmental stages (Fig. 1), two biological replicates were made at each stage. These cDNA libraries were subjected to pair-end reading with the Illumina HiSeq 2000 platform, generating from 56 million to 64 million pair-end raw reads of 100 bp in length, respectively. And the raw data have been submitted to SRA database of NCBI (Accession ID: SRP070860; Link: https://trace.ncbi.nlm.nih.gov/Traces/sra/?study=SRP070860). After removing the low-quality reads and trimming the adapter sequences, we obtained from 27 million to 31 million clean reads (Table 2). The subsequent analyses were based on the clean data. Though only two biological replicates were used for RNA-seq, the correlation analysis showed that the R^2^ of the two replicates of all the samples were greater than 0.95. It fully illustrated the consistency of two biological replicates and the reliability of the RNA-seq results.

**Table 2:** Summary of the sequencing data

Sample name	Raw reads	Clean reads	Clean bases	Error rate(%)	Q20(%)	Q30(%)	GC content(%)
B1A	56801854	54944048	6.86G	0.03	96.90	93.75	46.02
B1B	58642586	56945838	7.12G	0.03	97.00	93.94	45.84
B2A	60467584	58763160	7.34G	0.03	96.4	92.73	46.45
B2B	57458296	55118356	6.88G	0.03	96.23	92.42	47.42
B3A	56058442	54572136	6.82G	0.03	96.25	92.43	46.49
B3B	62161910	60249422	7.54G	0.03	96.32	92.57	46.41
W1A	58127846	56521424	7.06G	0.03	96.30	92.47	46.14
W1B	50809432	49850444	6.24G	0.03	96.40	92.59	46.59
W2A	60765156	59192440	7.40G	0.03	96.47	92.84	46.10
W2B	61258710	59500464	7.44G	0.03	96.54	92.96	46.53
W3A	64217138	61521590	7.70G	0.03	96.19	92.28	47.07
W3B	64263360	62332092	7.80G	0.03	96.23	92.35	46.76

A and B: two biological replicates of each stages; Raw reads: the original number of reads by sequencing; Clean reads: the number of reads after removing the low-quality reads and trimming the adapter sequences from raw reads; Clean bases: number of clean reads multiplied by length of clean reads. Error rate: the average base sequencing error rates; Q20 and Q30: Phred score, refer to the accuracy of sequenced bases were 99 and 99.9%; GC content: the percentage of G and C in total bases

### Sequence alignment and mapping to the reference genome

We used the French-Italian Public Consortium for Grapevine Genome Characterization, publically accessible version of the complete *V. vinifera* genome at 12× coverage (ftp://ftp.ensemblgenomes.org/pub/release-23/plants/fasta/vitis_vinifera/dna/) [58], as the reference genome. After quality control, the reads from black spine grape (64–74%) and white spine grape (63–76%) successfully aligned to the reference genome. Most of the reads from each fruit developmental stage for black and white spine grape aligned to a single position. These uniquely mapped reads account on average for approximately 67 and 71% of the total number of sequenced reads for the black and white spine grape, respectively. The number of reads from each fruit developmental stage for both black and white spine grape mapped to ‘+’ and ‘–’ strand were mostly equal. The number of non-splice reads was approximately twice to splice reads (Table 3).

**Table 3:** The clean reads mapped to the reference genome

Sample name	Total reads	Total mapped	Multiple mapped	Uniquely mapped	Reads map to ‘ + ’	Reads map to ‘-’	Non-splice reads	Splice reads
B1A	54944048	40808925 (74.27%)	794409 (1.45%)	40014516 (72.83%)	20002451 (36.41%)	20012065 (36.42%)	24600401 (44.77%)	15414115 (28.05%)
B1B	56945838	42479460 (74.6%)	859482 (1.51%)	41619978 (73.09%)	20805711 (36.54%)	20814267 (36.55%)	25634857 (45.02%)	15985121 (28.07%)
B2A	58763160	37649622 (64.07%)	748838 (1.27%)	36900784 (62.8%)	18441742 (31.38%)	18459042 (31.41%)	23233578 (39.54%)	13667206 (23.26%)
B2B	55118356	36141693 (65.57%)	728542 (1.32%)	35413151 (64.25%)	17717304 (32.14%)	17695847 (32.11%)	22963769 (41.66%)	12449382 (22.59%)
B3A	54572136	36055433 (66.07%)	800693 (1.47%)	35254740 (64.6%)	17628599 (32.3%)	17626141 (32.3%)	22458722 (41.15%)	12796018 (23.45%)
B3B	60249422	40232538 (66.78%)	961911 (1.6%)	39270627 (65.18%)	19632707 (32.59%)	19637920 (32.59%)	24890373 (41.31%)	14380254 (23.87%)
W1A	56521424	43356467 (76.71%)	856353 (1.52%)	42500114 (75.19%)	21230181 (37.56%)	21269933 (37.63%)	26214684 (46.38%)	16285430 (28.81%)
W1B	49850444	38208017 (76.65%)	751623 (1.51%)	37456394 (75.14%)	18713704 (37.54%)	18742690 (37.6%)	22835496 (45.81%)	14620898 (29.33%)
W2A	59192440	44324521 (74.88%)	750136 (1.27%)	43574385 (73.61%)	21759103 (36.76%)	21815282 (36.85%)	26852753 (45.37%)	16721632 (28.25%)
W2B	59500464	45258909 (76.06%)	808909 (1.36%)	44450000 (74.71%)	22202737 (37.32%)	22247263 (37.39%)	27353360 (45.97%)	17096640 (28.73%)
W3A	61521590	39182938 (63.69%)	793374 (1.29%)	38389564 (62.4%)	19130918 (31.1%)	19258646 (31.3%)	24966612 (40.58%)	13422952 (21.82%)
W3B	62332092	42273326 (67.82%)	830849 (1.33%)	41442477 (66.49%)	20661523 (33.15%)	20780954 (33.34%)	26550475 (42.6%)	14892002 (23.89%)

**Fig. 2:**
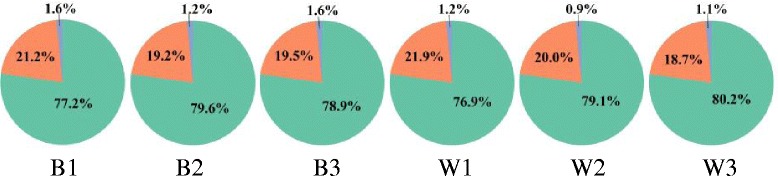
Percent of reads mapped to genome regions. The green part refers to percentage of reads mapped to exons; the orange part refer to percentage of reads mapped to intergenic regions; the blue part refer to percentage of reads mapped to introns

Approximately 80% of the reads from each fruit developmental stage for both black and white spine grape mapped to exons, 20% mapped to intergenic regions, and 1% mapped to introns (Fig. 2). The reads mapped to introns resulted from the residual of pre-mRNA and intron retention in the process of alternative splicing. However, the incomplete genome annotation led to the reads mapped to intergenic regions.

### Differential expression analysis

We used the RPKM value to show the gene expression level (Table 4). About 45–50% of the genes fall into the RPKM range of 0–1, 9–10% of the genes fall into the RPKM range of 1–3, 18–20% of the genes fall into the RPKM range of 3–15, 14–17% of the gens fall into the RPKM range of 15–60, and approximately 7% of the genes that RPKM value is greater than 60.

**Table 4:** The statistic of different gene expression level interval number

RPKM Interval	B1	B2	B3	W1	W2	W3
0 ~ 1	14793(45.10%)	16496(50.29%)	16410(50.03%)	15195(46.33%)	15234(46.45%)	16526(50.38%)
1 ~ 3	3220(9.82%)	2944(8.98%)	3061(9.33%)	3221(9.82%)	3253(9.92%)	2978(9.08%)
3 ~ 15	6771(20.64%)	5977(18.22%)	6174(18.82%)	6509(19.84%)	6347(19.35%)	6177(18.83%)
15 ~ 60	5668(17.28%)	5033(15.34%)	4961(15.12%)	5494(16.75%)	5389(16.43%)	4900(14.94%)
>60	2348(7.16%)	2350(7.16%)	2194(6.69%)	2381(7.26%)	2577(7.86%)	2219(6.77%)

Numbers of transcripts from the *V. vinifera* RefSeq dataset detected at various levels of abundance at each time-point, as calculated by reads per kilobase of exon per million reads (RPKM)

**Fig. 3:**
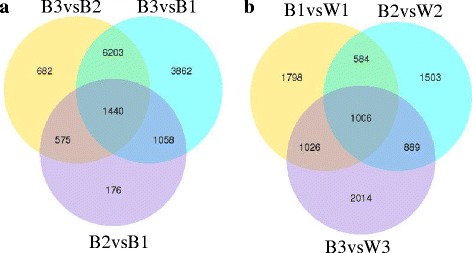
The venn diagrams of differential expression genes. Venn diagrams indicate the overlap of differential expression genes between B2vsB1, B3vsB1 and B3vsB2 (**a**), and between B1vsW1, B2vsW2 and B3vsW3 (**b**)

There were thousands of differential expression genes in three fruit developmental stages. But, we focused on the genes which were related to the change of berry skin color in fruit development. Thus, we compared the expression of genes in different stages, such as B2vsB1, B3vsB2, B3vsB1, B1vsW1, B2vsW2 and B3vsW3. Then, we used the differential expression genes which were gained from the comparison to make venn diagrams (Fig. 3). In the venn diagram of B2vsB1, B3vsB2 and B3vsB1, 1440 differential expression genes were found. And in the venn diagram of B1vsW1, B2vsW2 and B3vsW3, it was 1006. Accordingly, the scope of selecting the candidate differential expression genes is effectively narrowed.

### Selection of candidate genes

#### Functional enrichment analysis

**Fig. 4:**
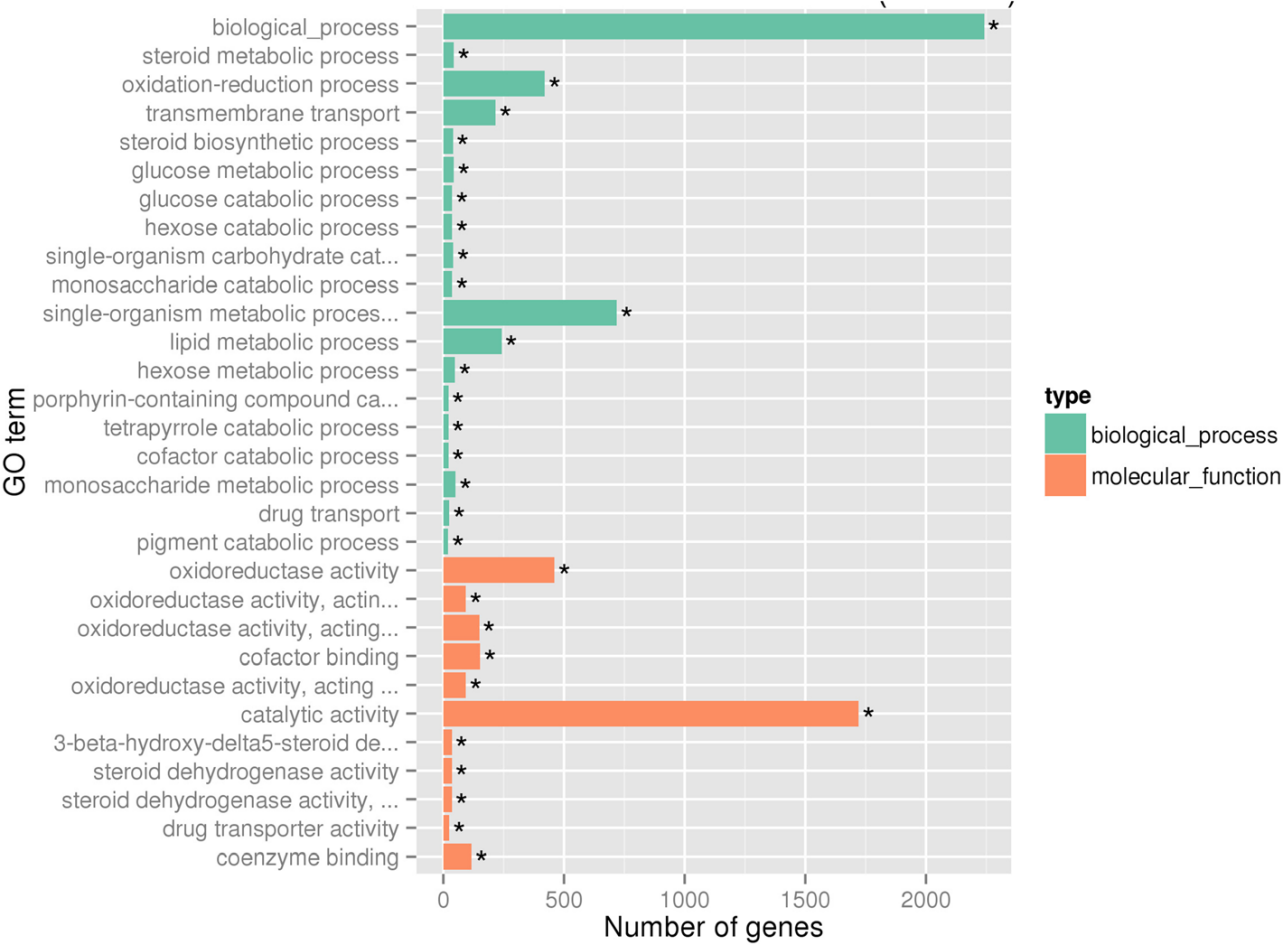
The most enriched GO terms (B2vsW2). Thirty most enriched GO terms were displayed, included 19 biological processes (*green*) and 11 molecular function (*orange*)

Gene Ontology (GO, http://www.geneontology.org/) is an international standardized gene function classification system which can be used to classify the function of the predicted genes. GO include three parts: molecular function, biological process and cellular component. We used the differential expression genes between B2 and W2 to make GO enrichment analysis. Thirty significant terms were found, which belonged to biological process and molecular function (Fig. 4). The color of the berry skin is determined by the pigment, and the anthocyanins are the main pigment in grapes [1, 2]. The anthocyanins were synthesized in the endoplasmic reticulum, and transported into vacuole by a serious of anthocyanins transporters [32]. Thus, in these 30 terms, transmembrane transport (GO:0055085) and pigment catabolic process(GO:0046149), which possibly contained the candidate genes, came into our attention. The transmembrane transport term contained 107 up-regulated expression genes and 110 down-regulated expression genes. The pigment catabolic process contained 6 up-regulated expression genes and 14 down-regulated expression genes (Additional file 1: Table S1).

**Fig. 5:**
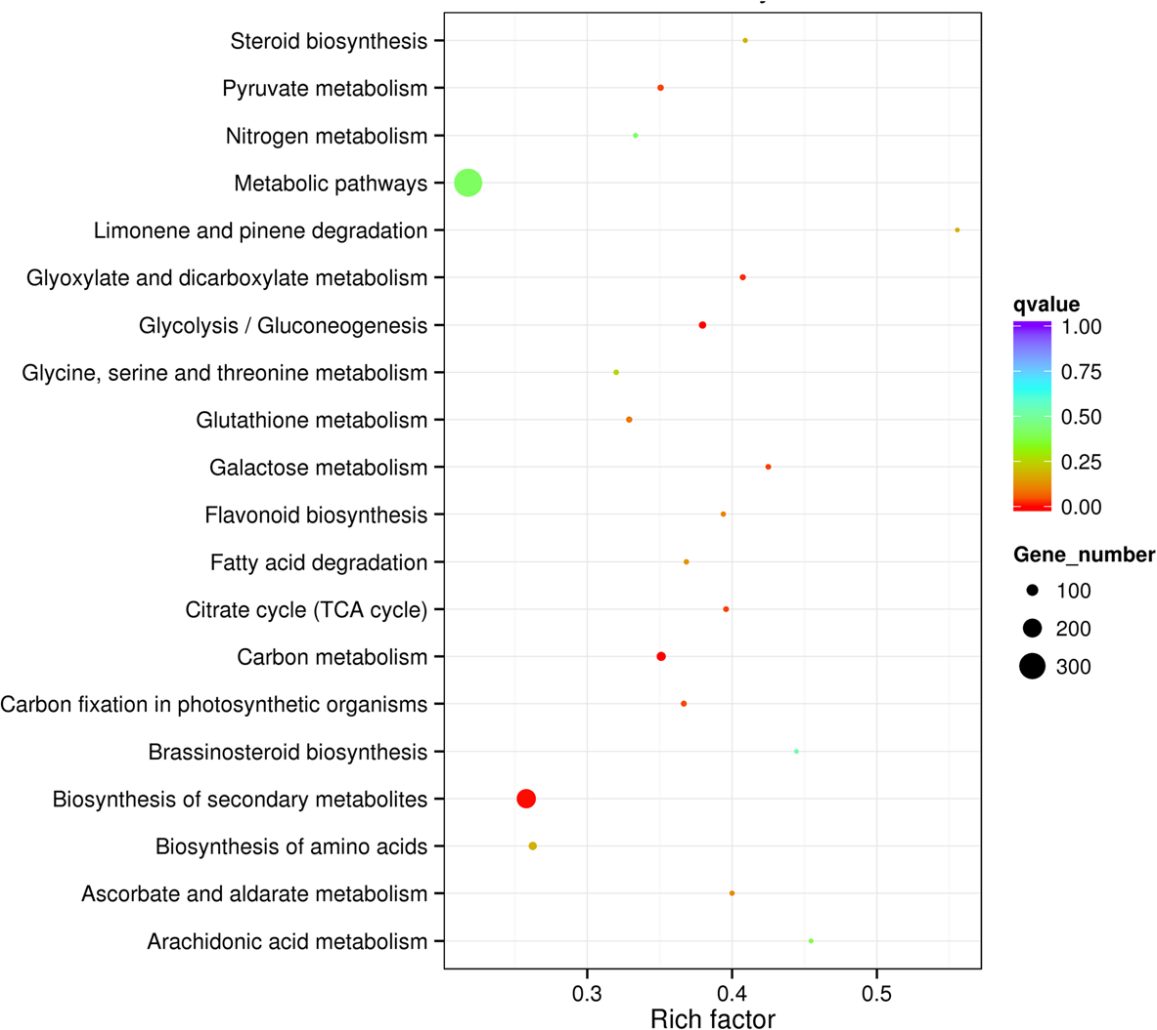
Statistics of KEGG pathway enrichment. The vertical axis refers to pathways, horizontal axis refers to rich factor. The sizes of the dots represent the numbers of differential expression genes in this pathway. The colors of the dots correspond to the scopes of Q-value

To identify the biological pathways activated in grape fruit, pathway enrichment was quantified in the KEGG database [59]. In grapes, anthocyanins synthesis is via the flavonoid pathways [10], which were significantly enriched in our study (Fig. 5). There were 33 background genes in the flavonoid biosynthesis pathway, 27 genes showed expressional differences (Table 5). In these 27 genes, VIT_00s0361g00040 was annotated as anthocyanidin reductase (ANR), VIT_01s0011g02960 and VIT_17s0000g04150 were annotated as leucoanthocyanidin reductase(LAR), which were related to the synthesis of proanthocyanidins [20, 21]. VIT_18s0001g03470 was annotated as flavonol synthase (FLS), which was related to the synthesis of flavonol aglycones [60, 61]. VIT_02s0033g00410 was annotated as transcription factor MYBA1, which can effectively regulate the anthocyanins biosynthesis [41–43]. VIT_02s0025g04720 and VIT_16s0039g02230 were respectively annotated as LDOX and UFGT, which were directly related to anthocyanins biosynthesis [14, 15, 22–25]. VIT_03s0063g00140, VIT_07s0031g00350, VIT_11s0016g02610 and VIT_12s0028g03110 were annotated as OMT, which participated in the methylation modification of anthocyanins [26, 27]. Other genes were related to the upstream pathways of the flavonoid biosynthesis.

**Table 5:** The differential expression genes in the KEGG pathway of flavonoid biosynthesis

Gene_ID	RPKM value						Annotation
	B1	B2	B3	W1	W2	W3	
VIT_02s0033g00410	0.04	140.49	127.82	0.00	0.00	7.97	MYBA1
VIT_00s0361g00040	2.79	1.66	0.63	2.92	1.88	0.67	ANR
VIT_01s0011g02960	5.94	1.75	0.42	1.85	0.70	0.78	LAR1
VIT_02s0025g04720	38.96	1659.75	1030.34	26.99	36.62	87.43	LDOX
VIT_03s0063g00140	0.09	0.30	7.61	0.44	1.01	4.66	OMT
VIT_04s0023g03370	6.98	561.89	230.96	3.71	2.35	6.48	F3H
VIT_05s0136g00260	8.42	4686.98	3990.83	5.62	4.05	41.12	CHS
VIT_06s0004g08150	23.71	109.70	79.78	29.92	18.20	42.08	Trans-cinnamate 4-monooxygenase
VIT_06s0009g02830	6.79	237.73	281.50	0.13	0.20	0.12	F3′5′H
VIT_06s0009g02860	0.05	6.73	1.89	0	0	0	F3′5′H
VIT_06s0009g03050	0	0.04	0.57	0	0	0	F3′5′H
VIT_07s0031g00350	1.15	448.44	1191.02	16.33	50.62	648.28	OMT
VIT_08s0040g00780	11.03	40.57	48.50	8.59	8.74	29.68	Cytochrome P450
VIT_11s0016g02610	1.04	1.64	1.03	0.07	0	0	OMT
VIT_11s0065g00350	0.02	0.57	0.60	0.05	0.82	0.10	Trans-cinnamate 4-monooxygenase
VIT_12s0028g03110	2.76	0.12	0.04	1.62	0.59	0.18	OMT
VIT_13s0067g03820	48.72	700.54	545.88	33.78	26.95	91.33	CHI
VIT_14s0068g00920	2.25	490.58	278.42	3.15	7.42	13.49	CHS
VIT_14s0068g00930	0.20	2.97	2.04	2.17	6.63	1.69	CHS
VIT_16s0039g02350	2.31	3.48	13.25	0.00	0.17	1.02	DFR
VIT_17s0000g04150	14.12	10.12	20.50	9.55	11.13	9.64	LAR2
VIT_17s0000g07200	6.74	47.12	57.77	12.97	17.55	53.45	F3′H
VIT_17s0000g07210	0.60	3.35	3.37	0.53	0.79	2.40	F3′H
VIT_18s0001g03470	0.02	0.00	0.03	0.07	10.20	0.39	FLS
VIT_18s0001g12800	4.86	35.81	24.90	7.04	7.24	9.41	DFR
VIT_16s0039g02230	0.04	1214.43	1009.98	0.08	0.04	0.16	UFGT
VIT_18s0001g14310	45.39	120.46	67.93	46.73	66.97	124.79	Flavanone 3-dioxygenase

Expression levels are shown in RPKM for each sample. The annotation was the results of the gene blast in Swiss-prot. *ANR* anthocyanidin reductase, *LAR* leucoanthocyanidin reductase, *LDOX* leucoanthocyanidin dioxygenase, *OMT O*-methyltransferase, *F3H* flavanone-3β-hydroxylase, *CHS* chalcone synthase, *F3′5′H* flavonoid-3′5′-hydroxylase, *CHI* chalcone isomerase, *DFR* dihydroflavonol 4-reductase, *F3′H* flavonoid-3′-hydroxylase, *FLS* flavonol synthase, *UFGT* UDP-glucose:flavonoid-3-*O*-glucosyltransferase;

#### Expression pattern analysis

**Fig. 6:**
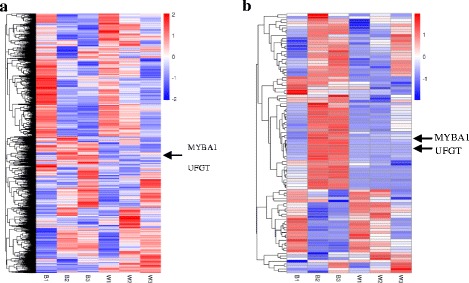
The heatmap of the differential expression genes. **a** The heatmap of all the differential expression genes. **b** The heatmap of the selected 100 differential expression genes. Using log_10_(RPKM + 1) value to cluster, the red color referred to high expressed genes, the blue color refer to low expressed genes. From blue to red, refer to the log_10_(RPKM + 1) value gradually rised up. The vertical axis refer to the genes showed similar expressed pattern were clustered in a group

We also used differential expression gene clustering methodology to find the genes that are related to the biosynthesis of anthocyanins in spine grape. The genes that showed same expression pattern were clustered together, and they may obtain the same function or belong to the same biological pathway. We used all of the differential expression genes to draw a heatmap (Fig. 6a). The genes that had the same or similar expression patterns were effectively clustered together. However, so many clusters were produced, and it was difficult to find the clusters that contained the genes closely related to the color of the berry skin. But, the previous studies have suggested that *UFGT*(VIT_16s0039g02230) [1, 24, 25] and *MybA1*(VIT_02s0033g00410) [41] were the key genes to regulate the synthesis of the anthocyanins. Thus, we selected the genes whose expression patterns were similar to these two genes. Then, we obtained 100 genes and used them to make a heatmap (Fig. 6b). The genes were divided into two groups that contained 68 and 32 genes respectively. And the expression pattern of the group that contained 68 genes was very similar to *UFGT* and *MybA1*. Then, we can select the genes that were related to the biosynthesis and transport of anthocyanins.

#### The candidate genes

**Fig. 7:**
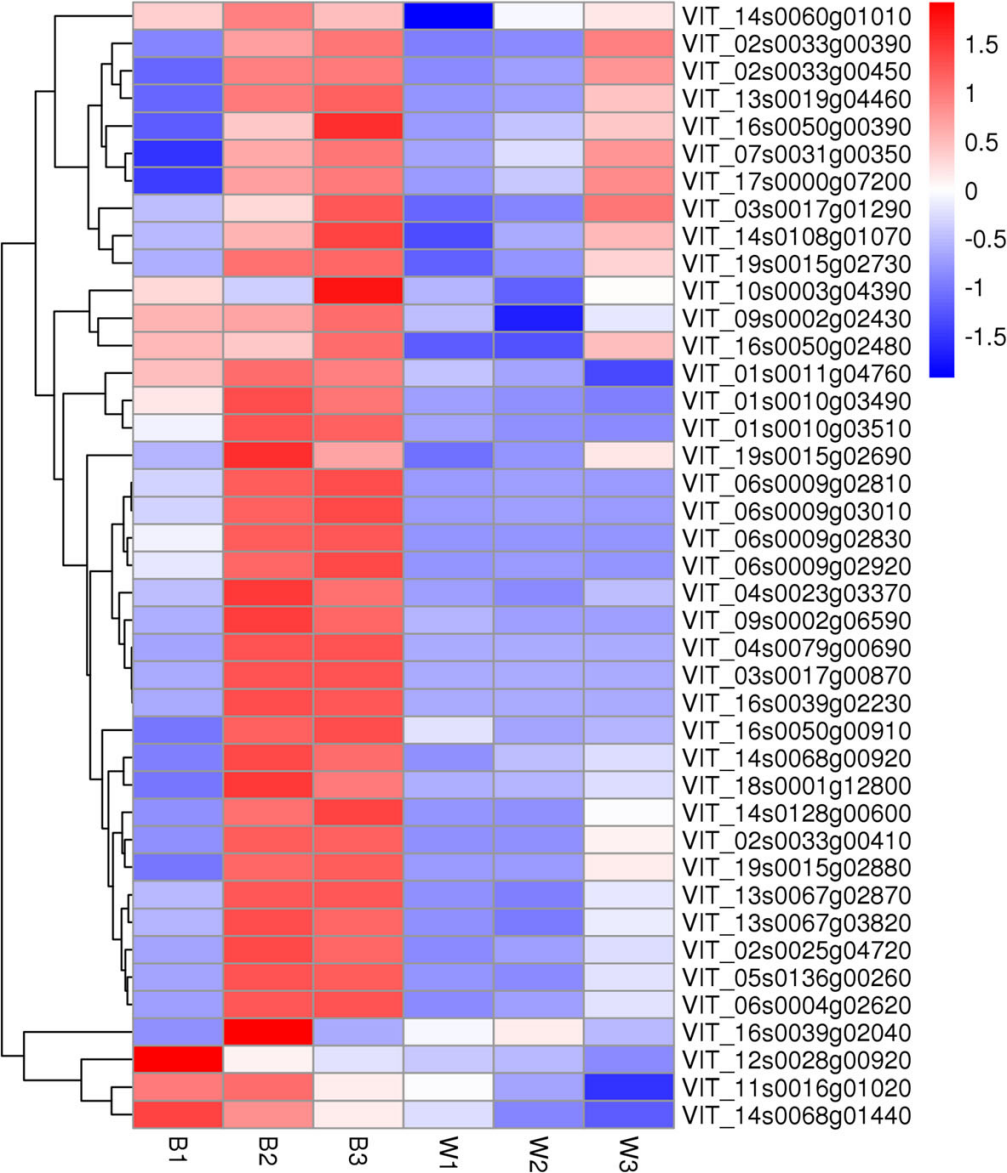
The heatmap of the 41 candidate genes. The denotations are the same as in Fig. 5. The corresponding gene IDs were noted on the right side of the picture

After these analyses, we finally obtained 41 differential expression genes, which were considered to be related to the biosynthesis and transport of anthocyanins in the berry skin of spine grape (Table 6). The expression patterns of these genes were very similar, mainly expressed in B2 and B3 (Fig. 7). In these candidate genes, just one gene (VIT_14s0128g00600) was not annotated. Yet its expression pattern was very similar to the other candidate genes, and we inferred that it was probably related to the anthocyanins accumulation.

**Table 6:** Expression profiles of anthocyanin biosynthesis and transport candidate genes in spine grape

Gene_ID	RPKM value						Annotation
	B1	B2	B3	W1	W2	W3	
VIT_02s0033g00410	0.04	140.49	127.82	0.00	0.00	7.97	MYBA1
VIT_02s0033g00390	0.03	128.67	322.83	0.00	0.16	248.31	MYBA2
VIT_02s0033g00450	0.00	358.15	409.69	1.21	2.49	222.68	MYBA3
VIT_01s0011g04760	97.59	124.81	117.63	67.22	61.35	46.54	MYB4
VIT_14s0060g01010	24.39	38.55	27.01	3.48	17.65	20.80	bHLH
VIT_14s0108g01070	3.26	11.77	29.28	0.91	2.80	11.50	NAC
VIT_06s0004g02620	0.93	202.54	219.54	0.40	0.88	5.22	PAL
VIT_13s0019g04460	4.30	232.02	340.47	8.63	10.27	89.01	PAL2
VIT_16s0039g02040	9.04	30.83	9.78	12.76	14.09	10.35	4CL
VIT_16s0050g00390	9.67	45.72	129.44	15.09	20.51	45.27	4CL
VIT_05s0136g00260	8.42	4686.98	3990.83	5.62	4.05	41.12	CHS3
VIT_14s0068g00920	2.25	490.58	278.42	3.15	7.42	13.49	CHS2
VIT_13s0067g03820	48.72	700.54	545.88	33.78	26.95	91.33	CHI1
VIT_13s0067g02870	132.01	654.60	660.10	99.54	89.00	184.20	CHI2
VIT_04s0023g03370	6.98	561.89	230.96	3.71	2.35	6.48	F3H1
VIT_11s0016g01020	62.59	74.70	15.97	11.77	3.55	0.21	F3′H
VIT_17s0000g07200	6.74	47.12	57.77	12.97	17.55	53.45	F3′H
VIT_06s0009g02830	6.79	237.73	281.50	0.13	0.20	0.12	F3′5′H
VIT_06s0009g03010	2.84	262.91	424.92	0.23	0.32	0.26	F3′5′H
VIT_06s0009g02810	1.28	45.46	55.50	0.00	0.10	0.04	F3′5′H
VIT_06s0009g02920	2.99	75.91	133.90	0.06	0.09	0.04	F3′5′H
VIT_18s0001g12800	4.86	35.81	24.90	7.04	7.24	9.41	DFR
VIT_02s0025g04720	38.96	1659.75	1030.34	26.99	36.62	87.43	LDOX
VIT_16s0039g02230	0.04	1214.43	1009.98	0.08	0.04	0.16	UFGT
VIT_09s0002g06590	0.55	511.11	202.65	0.68	0.00	0.10	5GT
VIT_07s0031g00350	1.15	448.44	1191.02	16.33	50.62	648.28	OMT
VIT_01s0010g03510	9.97	736.92	512.06	0.87	0.12	0.03	OMT1
VIT_01s0010g03490	7.95	79.79	44.64	0.56	0.24	0.00	OMT2
VIT_14s0068g01440	21.78	11.03	4.82	2.91	0.93	0.39	AT
VIT_03s0017g00870	0.00	246.75	227.53	0.02	0.00	0.02	3AT
VIT_12s0028g00920	48.17	23.64	20.90	19.40	18.62	16.04	GST3
VIT_04s0079g00690	0.03	3274.70	3166.24	0.22	0.06	0.14	GST4
VIT_19s0015g02690	8.25	238.49	58.89	3.48	5.57	26.74	GSTu25
VIT_19s0015g02730	5.81	84.17	96.10	1.79	4.27	27.43	GST5
VIT_19s0015g02880	21.32	192.30	211.83	27.78	28.43	70.32	GST
VIT_16s0050g00910	0.72	494.01	746.08	13.13	3.05	4.36	AM2
VIT_16s0050g02480	79.44	76.26	99.26	40.72	39.48	79.18	ABCC1
VIT_10s0003g04390	97.89	82.88	145.60	78.68	66.86	91.25	ABCC2
VIT_09s0002g02430	34.16	36.74	49.76	16.68	6.89	20.86	ABCC8
VIT_03s0017g01290	10.84	42.46	222.45	3.05	4.87	153.61	ABCG11
VIT_14s0128g00600	0.00	118.71	290.45	0.07	0.00	6.32	Unkown

Expression values are shown in RPKM for each sample. The annotation was the results of the gene blast in swiss-prot. *bHLH* basic helix–loop-helix proteins, *PAL* phenylalanine ammonia lyase, *4CL* 4-coumaroyl-coA synthase, *CHS* chalcone synthase, *CHI* chalcone isomerase, *F3H* flavanone-3β-hydroxylase, *F3′H* flavonoid-3′-hydroxylase, *F3′5′H* flavonoid-3′5′-hydroxylase, *DFR* dihydroflavonol 4-reductase, *LDOX* leucoanthocyanidin dioxygenase, *UFGT* UDP-glucose:flavonoid-3-*O*-glucosyltransferase, *5GT* UDP-glucose: anthocyanin 5-*O*-glucosyltransferase, *OMT O*-methyltransferase, *AT* anthocyanin acyltransferases, *GST* glutathione S-transferase, *AM* anthoMATEs, *ABCC* ATP binding cassette

#### Selected candidate *UGT* genes in white spine grape

Trace amount of anthocyanins were detected in white spine grape (Table 1). Thus, we inferred that some other *UGT*s are probably related to anthocyanins accumulation in white spine grape. We compared the third stage of white spine grape with the first stage, 62 significant differential expression genes were annotated as UGTs (Additional file 2: Table S2), containing 20 up-regulated and 42 down-regulated genes. From the 20 up-regulated genes, we selected the most 5 significant differential expression genes as the candidate genes (Table 7). All of these five candidate genes were above 4.5-fold higher in W3 than in W1. Especially, VIT_00s0324g00050 showed highly significant differential expression (*P* = 3.79E-129).

**Table 7:** The candidate *UGT* genes selected from comparison between W3 and W1

Gene_ID	Readcount_W3	Readcount_W1	Log2FoldChange	P-value	Blast swiss prot
VIT_17s0000g08100	214.94	0.00	-	1.41E-06	UDP-glycosyltransferase 90A1
VIT_00s0324g00070	27.64	0.43	5.9911	0.0001105	UDP-glycosyltransferase 85A2
VIT_00s0324g00050	9505.73	172.13	5.7873	3.79E-129	UDP-glycosyltransferase 85A2
VIT_06s0004g05780	45.61	0.87	5.714	4.10E-09	UDP-glycosyltransferase 76E2
VIT_08s0007g04570	382.63	15.45	4.6299	2.18E-46	UDP-glycosyltransferase 73D1

Log2FoldChange: log2 (Readcount_W3/ Readcount_W1); P-value was adjusted; Predicted amino acid sequence was used to blast in swiss prot, showing a high homology with Arabidopsis thaliana *UGT*s

#### qRT-PCR analysis

**Fig. 8:**
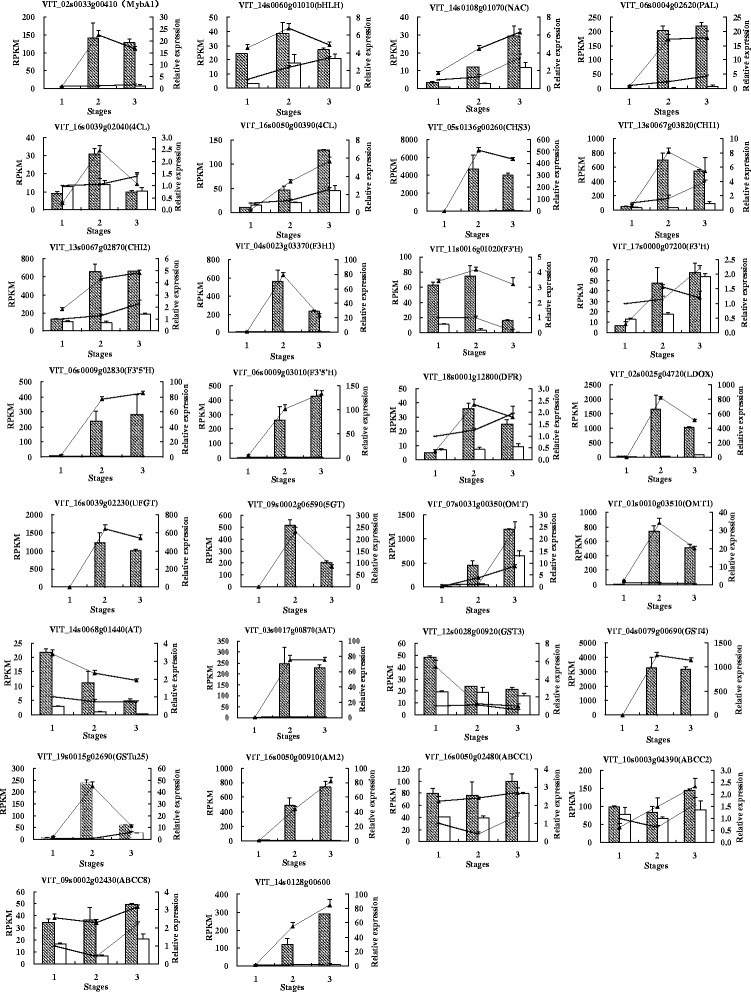
Quantitative real-time PCR validation of RNA-Seq data. Relative expression profiles of 30 genes showed the expression fold changes (FC) in comparison between the three fruit developmental stages in the black and white spine grapes. Histograms represent expression levels as assessed by RNA-Seq, data are reported as means ± SE of two biological replicates (*left axis*), the columns with diagonal lines or blank represent the RPKM of black or white spine grape, respectively. The line charts represent expression fold changes as assessed by qRT-PCR, data are reported as means ± SE of three replicates (*right axis*), the lines with black triangle or ‘×’ mark represent black or white spine grape, respectively

To confirm the results obtained from RNA-seq, relative expression profiles of 30 genes were analyzed by real-time RT-PCR at the three fruit developmental stages in black and white spine grapes. These 30 genes related to the biosynthesis and transport of anthocyanins were chosen for qRT-PCR analysis. The black spine grape showed much higher gene expression of the selected genes than that of the white spine grape. For most of the genes, the qRT-PCR results were consistent with those obtained from the expression profile determined from RNA-Seq data (Fig. 8).

## Discussion

### Anthocyanin composition and content in black and white *V. davidii*

In *V. vinifera*, only 3-*O*-monoglucoside anthocyanins were detected [6]. However, in our research, 3,5-*O*-diglucoside anthocyanins was the predominant anthocyanins in *V. davidii*. The studies have shown that, the 3,5-*O*-diglucoside anthocyanins were usually detected in other Chinese wild grapes [5, 7, 55, 56]. Therefore, the *V. davidii* was closer to the other Chinese wild grapes from the perspective of the composition of anthocyanins. We inferred that the high 3-5-*O*-disglucoside anthocyanins concentration in black spine grape was closely related to the expression of VIT_09s0002g06590 (*5GT*).

Interestingly, we also detected trace amount of anthocyanins in white *V. davidii*. It was generally accepted that anthocyanins were present only in red grapes, and it was used as a standard to define the difference between red grape and white grape [46, 62]. But, a very recent research suggested that some white grapes contained measurable traces of anthocyanins [63]. Previous researches showed that *UFGT* gene was not expressed in white grape berries, and the expression of *UFGT* was regulated by two very similar regulatory genes, *VvMYBA1* and *VvMYBA2*, which were not transcribed in white grape berries [46, 62]. The present study revealed that *UFGT* (VIT_16s0039g02230) did not express in white *V. davidii*. However, why did we detect anthocyanins in white grape berries? A previous study showed that there were as many as 181 putative *UDP-glycosyltranferases* (*UGT*s) found in the genome of *V. vinifera* [64]. Arapitsas et al. [65] detected measurable trace amounts of anthocyanins in some white grape varieties, and they inferred that the other *UGT*s expressed in berry skin accepts anthocyanidin as substrate and is therefore involved in the synthesis of trace amount anthocyanins detected in the berry skin of white grape. In our study, 5 candidate *UGT* genes were above 4.5-fold higher in W3 than in W1, especially VIT_00s0324g00050 showed highly significant differential expression (*P* = 3.79E-129). These genes are probably related to anthocyanins accumulation in white *V. davidii*.

### The candidate genes related to anthocyanins biosynthesis

**Fig. 9:**
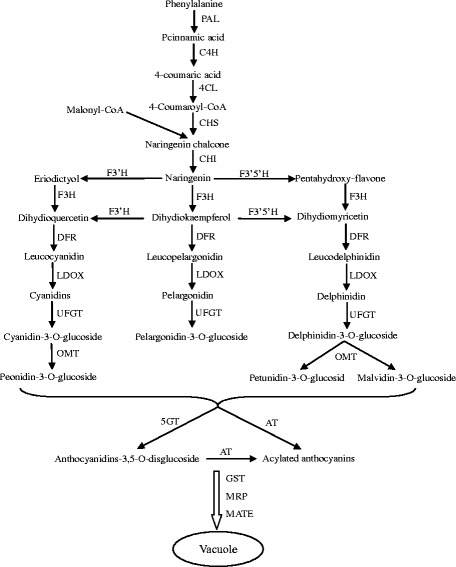
The diagram of the anthocyanins biosynthetic pathway. Abbreviations for the enzymes: PAL phenylalanine ammonia lyase, C4H cinnamate-4-hydroxylase, 4CL 4-coumaroyl-coA synthase, CHS chalcone synthase, CHI chalcone isomerase, F3′H flavonoid-3′-hydroxylase, F3′5′H flavonoid-3′5′-hydroxylase, F3H flavanone-3β-hydroxylase, DFR dihydroflavonol 4-reductase, LDOX leucoanthocyanidin dioxygenase, UFGT UDP-glucose:flavonoid-3-*O*-glucosyltransferase, OMT *O*-methyltransferase, 5GT UDP-glucose:anthocyanin-5-*O*- glucosyltransferase; MRP multidrug resistance-associated protein, MATE toxic compound extrusion, AT anthocyanin acyltransferases, GST glutathione S-transferase

The synthesis of anthocyanins via the phenylpropanoid and flavonoid pathway, and it is catalyzed by a serious of enzymes (Fig. 9) [1, 10, 37]. However, most of the genes coding for these enzymes are multi-copied in the grape genome [65]. Phenylalanine ammonia lyase (PAL) catalyzes the first step of the phenylpropanoid pathway. In our study, two differential expression genes (VIT_06s0004g02620 and VIT_13s0019g04460) were annotated as PAL. VIT_06s0004g02620 was mainly expressed in B2 and B3, but just had a trace amount expression in W3. VIT_13s0019g04460 was also mainly expressed in B2 and B3, and had a high expression in W3. Thus, we inferred that VIT_06s0004g02620 played a major role in the pathway. Under the action of C4H and 4CL, 4-Coumaroyl-CoA was produced. We did not find any obvious differential expression genes annotated as C4H. However, VIT_16s0039g02040 and VIT_16s0050g00390 were annotated as 4CL. Chalcone synthase (CHS) is the first committed enzyme of the flavonoid pathway. Previous studies suggested that 3 *CHS* genes (*CHS1*, *CHS2* and *CHS3*) played a role in this pathway. Of the *CHS*s, the mRNAs of *CHS1*and *CHS2* were detected in both leaves and berry skins of white and red grape cultivars, whereas the mRNA of *CHS3* was mainly accumulated in the berry skin of red cultivars during coloration [65–67]. In our study, only *CHS2* (VIT_14s0068g00920) and *CHS3* (VIT_05s0136g00260) showed significant expression differences between black and white spine grapes. Especially, *CHS3* (VIT_05s0136g00260) was detected as having a very high expressional level in B2 and B3, and significantly higher than that in white spine grape. It was consistent with the previous studies [65–67]. Two *CHI* genes (VIT_13s0067g03820 and VIT_13s0067g02870) showed obvious differential expression levels between black and white spine grapes. These two *CHI* genes (*CHI1* and *CHI2*) have been reported by previous researches [65, 67, 68]. The researches suggested that two *F3H* genes (*F3H1* and *F3H2*) played a role in the flavonoid pathway, and the mRNA of *F3H2* was detected at a high level in grape berry skin during coloration [65, 67]. However, in our study, only the expression of *F3H1* (VIT_04s0023g03370) in black spine grape showed significantly higher than white spine grape. Maybe *F3H*s play different roles in the flavonoid pathway between *V. vinifera* and *V. davidii*. F3′H and F3′5′H belong to the cytochrome P450 super family, and catalyze hydroxylation at the 3′ and 3′,5′ positions of the B-ring of the flavonoid to produce the precursors for cyanidin-based anthocyanins and delphinidin-based anthocyanins, respectively. Thus, components of anthocyanins in grape berry skins are closely related to the expression of *F3′H* and *F3′5′H* [62, 69]. The cDNAs of *F3′H* and *F3′5′H* were first isolated from petunia [70, 71]. The previous studies showed that grapevines contain two copies of *F3′H* and sixteen copies of *F3′5′H*, both of the *F3′H*s are located on chr17, fifteen of *F3′5′H*s located on chr6, and one of *F3′5′H*s located on chr8 [72]. The previous study showed that, the mRNA levels of *F3′H* and *F3′5′H* were high in grape berry skins at the harvest stage [73]. In our study, two (VIT_11s0016g01020 and VIT_17s0000g07200) and four (VIT_06s0009g02830, VIT_06s0009g03010, VIT_06s0009g02810 and VIT_06s0009g02920) differential expression genes were annotated as F3′H and F3′5′H, respectively. Of the two *F3′H*s, one *F3′H* gene (VIT_17s0000g07200) was located on chr17. However, the other *F3′H* gene (VIT_11s0016g01020) was located on chr11. In addition, VIT_11s0016g01020 showed more significantly differential expression level than VIT_17s0000g07200 between black and white grapes. We suggested that VIT_11s0016g01020 (*F3′H*) played a major role in anthocyanins accumulation in black spine grape. All of the four *F3′5′H* genes showed high expression level in B2 and B3, especially VIT_06s0009g02830 and VIT_06s0009g03010. But *F3′5′H* genes were almost not expressed at any of the three stages in white spine grape. Besides, we could see that the expression level of *F3′5′H* was obviously higher than *F3′H* in black spine grape. This led to more pentahydroxy-flavone and leucodelphinidin production, which were substrates of malvidin-based anthocyanins. Thus, we detected malvidin-based anthocyanins as the predominant kind of anthocyanins in black spine grape. The dihydroflavonols were catalyzed by DFR and LDOX to produce anthocyanidin. We found the differential expression genes (VIT_18s0001g12800 and VIT_02s0025g04720) annotated as DFR and LDOX, which was consistent with previous studies [67, 68, 74, 75].

### Regulation of anthocyanins synthesis

It has been suggested that there was a *mybA* gene cluster on chromosome 2 of the grape, which contained *MYBA1*, *MYBA2* and *MYBA3* [48]. The expression of *MYBA1* and *MYBA2* could promote the synthesis of anthocyanins through regulating the expression of *UFGT* [41, 46, 47]. In our study, *MYBA1* (VIT_02s0033g00410) showed high expression levels in B2 and B3, but it did not express in white spine grape. *MYBA2* (VIT_02s0033g00390) and *MYBA3* (VIT_02s0033g00450) showed high expression levels in B2, B3 and W3. Jiao et al. [57] detected *Gret1* in white-fruited varieties of *V. davidii*. Thus, it was reasonable that *MYBA1* did not express in white spine grape. *MYBA2* could also promote the expression of *UFGT* [41]. Though *MYBA2* showed high expression level in W3, *UFGT* (VIT_16s0039g02230) almost did not express and just trace amount anthocyanins were detected in white spine grape. We could infer from this observation that *MYBA1* may play a major role in regulating the synthesis of anthocyanins in spine grapes. Besides, another *R2R3-MYB* gene VIT_01s0011g04760 was annotated as *MYB4* expressed both in black and white spine grapes. But it showed significantly differential expression between them. The expression level of VIT_01s0011g04760 in black spine grape was obviously higher than in white spine grape. Thus, it can be regarded as a new candidate gene and needs further studies to confirm whether this gene regulates the biosynthesis of anthocyanins in grapes.

The flavonoid biosynthesis pathway is under the control of Myb transcriptional factors, basic helix–loop-helix proteins (bHLH) and WD40-like proteins [7, 10, 17–19, 36, 38]. The first *bHLH* was submitted as *VvMYCA1* (accession number EF193002; gene ID VIT_15s0046g02560) [76, 77], which was considered to regulate the expression of *UFGT*. Subsequently, the second *bHLH* was called *VvMYC1* (accession number EU447172; gene ID VIT_07s0104g00090) [39], which was characterized as a component of the transcriptional complex regulating anthocyanin biosynthesis in grapevine. However, both of these two genes did not show obvious differences at the transcriptional level between black and white spine grapes in our study. But, another gene (VIT_14s0060g01010) annotated as bHLH exhibited differential expression between black and white spine grapes. It was shown that *WDR1* (Genbank accession number DQ517914) contributed positively to the accumulation of anthocyanins [77]. However, we did not find any *WD40* gene having obvious differential expression. In recent years, researches showed that NAC TFs were involved in the regulation of anthocyanins accumulation. A NAC TF has been proposed to be involved in the regulation of anthocyanins accumulation during the response of blood orange to cold exposure [78]. In addition, PpNAC1 can activate the transcription of *PpMYB10.1*, resulting in anthocyanins pigmentation in blood-fleshed peach [79]. In grape, there was no report that the NAC TFs were related to the anthocyanins accumulation. In our study, a candidate gene (VIT_14s0108g01070) was annotated as NAC, which may be related to anthocyanins accumulation in spine grapes.

### Modification of anthocyanins

Anthocyanidins need to be modified by glycosylation, methylation and acylation to form stabilized anthocyanins. In *V. vinifera*, the glycosylation was catalyzed by UDP-glucose: anthocyanidin: flavonoid glucosyltransferase (UFGT) at C3 position, and the gene encoding UFGT had been cloned [24, 80]. The action of UFGT was crucial for anthocyanin accumulation in grape berry skin [1, 22, 23, 25, 50, 67, 81, 82]. In our study, a differential expression gene (VIT_16s0039g02230) was annotated as UFGT, which had a high expression level in B2 and B3, and did not express in white spine grape. However, in non-*V. vinifera* species, the 3-5-*O*-disglucoside anthocyanins are widely present [7, 8]. There must be another UDP-glucose: anthocyanidin: flavonoid glucosyltransferase to catalyze glycosylation at C5 position. In recent years, a few studies reported the isolation of *5GT* genes in grapes [83, 84]. Jánváry et al. [83] cloned functional *Cha5GT* and nonfunctional *Dia5GT* from the heterozygous hybrid cultivar ‘Regent’, a cross of *V. vinifera* cv. ‘Diana’ and the interspecific hybrid cv. ‘Chambourcin’. The functional analysis of *Cha5GT* and *Dia5GT* suggested that two mutations in the *5GT* gene eliminated its enzymatic activity. Because of the absence of active *5GT*, dis-glucosidic anthocyanins could not be produced in *V. vinifera* red grapes. He et al. [84] cloned the full-length cDNA of UDP-glucose: anthocyanin 5-*O*-glucosyltransferase (*Va5GT*) from *V. amurensis* Rupr. cv. ‘Zuoshanyi’. The results suggested that Va5GT was a key enzyme in the biosynthesis of dis-glucosidic anthocyanins in *V. amurensis* grape berries. In our study, a differential expression gene (VIT_09s0002g06590) showed a high expressional level just in B2 and B3, which was annotated as UGT. It has high homology with *Va5GT*. Thus, we inferred that the expression of VIT_09s0002g06590 (*5GT*) led to a high 3-5-*O*-disglucoside anthocyanins concentration in black spine grape.

In plants, methylated anthocyanidins accounted for a large proportion of the total reported anthocyanidins [3]. Especially, three methylated anthocyanidins, peonidin, petunidin and malvidin, were commonly present in grape berry skin. The methylation was catalyzed by S-adenosyl-L-methionine (SAM) or *O*-methyltransferase (OMT) at the C3 positions or both at the C3 and C5 positions on the B rings of the anthocyanins in grape [26, 27]. And the cDNAs of several OMT had been cloned in grapes [85, 86]. Subsequently, a QTL for anthocyanins methylation variation was identified that was colocalized with a cluster of three putative *OMT* genes: VIT_01s0010g03470 (*OMT3*), VIT_01s0010g03490 (*OMT2*) and VIT_01s0010g03510 (*OMT1*) [87, 88]. Fournier-Level et al. [87] reported that *OMT2* gene presented two SNPs associated with methylation level. It probably led to a structural change of the OMT2 protein, thus significantly affected the enzyme specific catalytic efficiency for the 3′-*O*-methylation of delphinidin-3-glucoside. In this study, three differential expression genes (VIT_07s0031g00350, VIT_01s0010g03510 and VIT_01s0010g03490) were annotated as OMTs. Of the three genes, VIT_01s0010g03510 (*OMT1*) and VIT_01s0010g03490 (*OMT2*) expressions were consistent with the previous researches [87, 88], which did not express in white spine grape. It is interesting that *OMT1* showed higher activity than *OMT2* in black spine grape. In addition, a new candidate gene (VIT_07s0031g00350) was also annotated as OMT, and showed high expressional level in B2 and B3. It probably played an important role in the methylation of anthocyanins in spine grape berry skin.

Acylation was a common modification of anthocyanins in grapes. It not only increased diversity of anthocyanins, but also improved the color stabilization and intensity for the anthocyanins [28]. Two enzyme families (BAHD-ATs and SCPL-ATs) have been reported as related to the acylation of anthocyanins [30, 31]. A recent study identified a number of QTLs associated with variation in acylated anthocyanin levels in F1 progeny from a ‘Syrah’x‘Pinot Noir’ cross. The strongest candidate genes within these QTLs included those belonging to the BAHD and SCPL acyltransferase family [88]. But, no QTL was found to cause the presence/absence of acylation in berries. In our study, two differential expression genes (VIT_14s0068g01440 and VIT_03s0017g00870) were annotated as acyltransferase (AT). Especially VIT_03s0017g00870 did not express in white spine grape and at the first stage of black spine grape. Yet it showed high expressional level in B2 and B3. Simultaneously, we detected acylated anthocyanins in black spine grape. Thus, this candidate gene can be very possibly related with the acylation of anthocyanins. It is known that *V. vinifera* cv. Pinot Noir does not synthesise acylated anthocyanins [89]. After SNP analysis of the candidate genes, at the position 36 of the putative coding region of VIT_03s0017g00870, A is replaced by G, compared with the PN40024 grapevine genome [58], but it does not alter the predicted amino acid sequence. Rinaldo et al. [90] identified a gene, anthocyanin 3-*O*-glucoside-6-*O*-acyltransferase (*Vv3AT*), which encoded a BAHD acyltransferase protein. This protein can promote the synthesis of acylated anthocyanins, which was transcriptionally regulated by *VvMYBA*. This is consistent with our study.

### Anthocyanins transport

Anthocyanins are synthesized in the endoplasmic reticulum and then transported into the vacuoles. Three kinds of anthocyanin transporters: glutathione S-transferase (GST), multidrug resistance-associated protein (MRP) and multidrug and toxic compound extrusion (MATE), were reported as related to the anthocyanins transport [32, 91]. In grapes, a few candidate anthocyanin transporters have been reported.

**Fig. 10:**
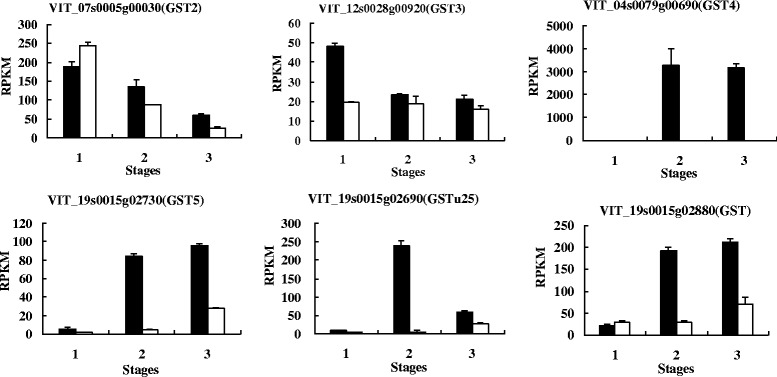
Expressional levels of six *GST*s in black and white spine grapes. The vertical axes refer to expression levels as assessed by RNA-Seq, horizontal axes refer to three berry developmental stages. The black columns represent black spine grape, the white columns represent white spine grape

Conn et al. [92] cloned five *GST*s (*VvGST1*,*VvGST2*,*VvGST3*,*VvGST4* and *VvGST5*) from *V. vinifera*. cv. Gamay Fréaux and the study showed that *VvGST1* and *VvGST4* coded for the enzymes which had the function of anthocyanins transport. However, in our study, *GST1* (VIT_19s0093g00320) was not detected, *GST4* (VIT_04s0079g00690) and *GST5* (VIT_19s0015g02730) showed significantly differential expression between black and white spine grape. Especially, *GST4* just express in B2 and B3 (Fig. 10). *GST2* (VIT_07s0005g00030) showed the highest expressional level in the first stage, and it possessed higher transcriptional level in white spine grape than black one. *GST3* (VIT_12s0028g00920) also showed the highest expressional level at the first stage, but did not show obvious differential expression between black and white spine grapes. Thus, we inferred that *GST4* (VIT_04s0079g00690) and *GST5* (VIT_19s0015g02730) play an important role in anthocyanins transport in spine grapes. Besides, another two candidate genes (VIT_19s0015g02690 and VIT_19s0015g02880) were annotated as GSTs, which showed higher expressional level in black spine grape than in white one (Fig. 10). Hence, these two candidate genes are also probably related to anthocyanins transport in spine grapes.

**Fig. 11:**
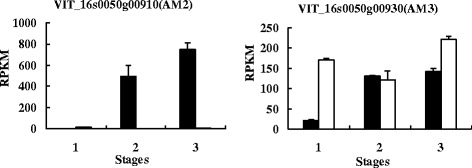
Expressional levels of *AM2* and *AM3* in black and white spine grapes. The denotations are the same as in Fig. 10

Gomez et al. [93] identified three multidrug and toxic compound extrusion (*MATE*) genes as candidate *anthoMATE*s(*AM*s). In their study, just *AM1* and *AM3* were cloned from *V. vinifera* L. cv. Syrah, *AM2* was not successfully cloned. They inferred that *AM2* was probably not expressed at detectable levels in mature berry or was a pseudogene. Accordingly, their results revealed that AM1 and AM3 just could transport acylated anthocyanins in the presence of Mg ATP [93]. Yet in our study, *AM1* (VIT_16s0050g00900) was hardly expressed in spine grapes, the expressional level of *AM3* (VIT_16s0050g00930) in white spine grape was higher than in black spine grape. However, *AM2* (VIT_16s0050g00910) was mainly expressed in B2 and B3 (Fig. 11). Thus, we infer that *AM2* plays an important role in spine grape anthocyanins transportation.

**Fig. 12:**
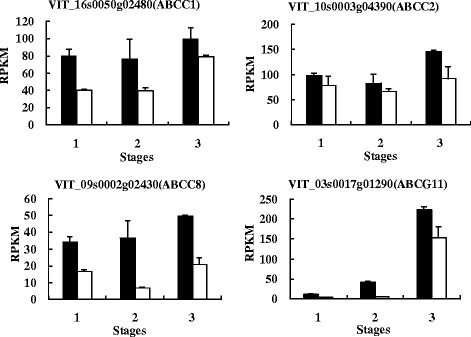
The expressional level of the *ABC*s in black and white spine grapes. The denotations are the same as in Fig. 10

The plant ATP binding cassette (ABC) transporters, in particular from the ABCC subfamily (formerly named multidrug resistance proteins [MRPs]) had been reported as related to the accumulation of flavonoid in vacuoles [94]. In grapes, an ABC transporter, ABCC1 was identified, which localizes to the tonoplast and is involved in the transport of glucosylated anthocyanidins [95]. In our study, four candidate genes were annotated as ABC transporters: VIT_16s0050g02480 (*ABCC1*), VIT_10s0003g04390 (*ABCC2*), VIT_09s0002g02430 (*ABCC8*) and VIT_03s0017g01290 (*ABCG11*). Francisco et al. [95] were unsuccessful to clone the *ABCC2* from the exocarp of grape, and inferred it might not be expressed in detectable amounts during the ripening stage. However, our study showed that both *ABCC1* and *ABCC2* were expressed in black and white spine grape berry skins. All of the four candidate ABCs were higher in expression in black spine grape than in white spine grape, but the differences were not very significant (Fig. 12). Thus, more studies are needed to confirm the key ABC transporters in grapes.

## Conclusions

*V. davidii* is the only Chinese wild grape species which possesses white berry varieties. High levels of 3,5-*O*-diglucoside anthocyanins were detected in the black berry skin of *V. davidii*. The present study investigated the transcriptome profiles of the berry skin from black and white spine grapes at three fruit developmental stages by Illumina mRNA-Seq technology. The examination of absolute expression count for every gene has enabled us to carry out a global investigation of gene expression at these three key time-points in black and white spine grapes. The transcriptome analysis presented thousands of DEGs. We used gene clustering and the enrichment of GO and KEGG to describe the transcriptional patterns of genes involved in anthocyanins accumulation. We found 41 differentially expressed genes probably related to anthocyanins accumulation in *V. davidii*, including the genes that encode enzymes, transcription factors and transporters involved in anthocyanins biosynthesis, regulation and transport. Some genes were consistent with the previous studies in other grape species, some were newly found in our study. Trace amounts of anthocyanins were detected in berry skins of white *V. davidii*. Five candidate *UGT* genes were probably related to the biosynthesis of anthocyanins in white *V. davidii*. In conclusion, the present study provides new insights into the understanding of anthocyanins accumulation in grapes.

## Methods

### Sample collection

Berries of black and white spine grapes (*V. davidii*) were used in this study. Both of the black and white spine grapes were collected from the wild in Hunan, China. The plants were grown in China National Germplasm Resources Repository of Grape, Zhengzhou Fruit Research Institute, Chinese Academy of Agricultural Sciences. Skins of grape berries were collected at three different fruit developmental stages: before veraison (Veraison refers to the stage that the berries begin to color and be soft) (40 days after flowering [DAF]), at veraison (80 DAF) and fruit ripe period (120 DAF) (Fig. 1). Two biological replicates were made at each stage. At each stage, we harvested 6–8 clusters at each sampling date, and 5 berries were collected from each cluster. Thus, 30–40 berries were used to collect the skin at each sampling date. After collection, samples were flash-frozen in liquid nitrogen and stored at −80 °C until further processing.

### The extraction and determination of anthocyanins

Extraction of total anthocyanins was as described by He et al. [7] with some modifications. The peels were put into mortars and ground in liquid nitrogen. Aliquots of 0.5 g ground powder were added into 10 ml centrifuge tubes with 8 ml 2% formic acid-methanol solution. After ultrasonic oscillation for 10 min, the extracts were put on a shaker in the dark in the table concentrator at 25 °C, 200 rpm for 30 min, followed by centrifugation at 4 °C, 12000 rpm for 10 min. The supernatants were transferred into 50 ml centrifuge tubes. The residues were re-extracted 3 times. The organic fractions were pooled, evaporated by a vacuum rotary evaporator (BUCH, USA) at 40 °C. The residual parts were poured into activated solid phase extraction cartridges. The solid phase extraction cartridges were washed with 5 ml water for 2 times. After removing the leacheate, the solid phase extraction cartridges were eluted with 10 ml methanol for 2 times. The filtrates were collected and evaporated to dryness, then re-dissolved in 5 mL 0.5% hydrochloric acid- methanol solution. Finally, the solutions were filtered through a 0.22 μm Millipore filter for analysis.

The anthocyanins content was determined by an ACQUITY Ultra Performance Liquid Chromatography (UPLC) system (Waters, Milford, MA, USA) linked to both a PDAeλ photo diode array detector (Waters, Milford, MA, USA) and a Micromass Quattro micro^TM^ API benchtop triple quadrupole mass spectrometer (Waters MS Technologies, Manchester, UK), with a electrospray ionization (ESI) source operating in multiple reaction monitoring (MRM) mode. Sample solutions were injected into a ACQUITY UPLC®HSS T3 column (2.1 × 150 mm i.d,with 1.8 μm particle size, Waters, Milford, MA, USA), which was maintained at 30 °C. The mass spectrometric acquisition parameters were as follows: ESI source temperature 150 °C, desolvation gas temperature 400 °C, desolvation gas flow rate 800 L/h, cone gas flow rate 50 L/h, collision gas (high purity argon gas) flow rate 0.14 mL/min. The mobile phase had acetonitrile as solvent A, and 0.5% hydrochloric acid solution as solvent B. The gradient profile began with 5–10% A for 1 min, 10–25% A for 16 min, 25–40% A for 18 min, 40–100% A for 19 min, and then returned to initial conditions for 20 min and for 5 min. The flow rate was 1.0 ml min^−1^ and the column temperature was set at 40 °C. The injection volume was 2.0 μl. The detection wavelength was 520 nm.

### Total RNA extraction and qualification

Total RNA was extracted using TIANGEN RNAprep Pure Plant Kit (Tiangen Biotech Beijing, China). RNA degradation and contamination was monitored on 1% agarose gels. RNA purity was checked using the NanoPhotometer® spectrophotometer (IMPLEN, CA, USA). RNA concentration was measured using Qubit® RNA Assay Kit in Qubit® 2.0 Flurometer (Life Technologies, CA, USA). RNA integrity was assessed using the RNA Nano 6000 Assay Kit of the Bioanalyzer 2100 system (Agilent Technologies, CA, USA).

### cDNA library construction and transcriptome sequencing

A total amount of 3 μg RNA per sample was used as the input material. Sequencing libraries were generated using NEBNext® Ultra™ Directional RNA Library Prep Kit for Illumina® (NEB, USA) following manufacturer’s recommendations and index codes were added to attribute sequences to each sample. Briefly, mRNAs were purified from the total RNAs using poly-T oligo-attached magnetic beads. Fragmentation was carried out using divalent cations under elevated temperature in NEBNext First Strand Synthesis Reaction Buffer (5X). First strand cDNA was synthesized using random hexamer primer and M-MuLV Reverse Transcriptase (RNaseH^−^). Second strand cDNA synthesis was subsequently performed using DNA Polymerase I and RNase H. In the reaction buffer, dNTPs with dTTP were replaced by dUTP. The remaining overhangs were converted into blunt ends via exonuclease/polymerase activities. After adenylation of 3′ ends of DNA fragments, NEBNext Adaptor with hairpin loop structure were ligated for hybridization. In order to select cDNA fragments of preferentially 150–200 bp in length, the library fragments were purified with AMPure XP system (Beckman Coulter, Beverly, USA). Then 3 μl USER Enzyme (NEB, USA) was used with size-selected, adaptor-ligated cDNAs at 37 °C for 15 min followed by 95 °C for 5 min before PCR. Then PCR was performed with Phusion High-Fidelity DNA polymerase, universal PCR primers and Index (X) Primer. The PCR products were purified (AMPure XP system) and library quality was assessed on the Agilent Bioanalyzer 2100 system.

The clustering of the index-coded samples was performed on a cBot Cluster Generation System using TruSeq PE Cluster Kit v3-cBot-HS (Illumia) according to the manufacturer’s instructions. After cluster generation, the library preparations were sequenced by an Illumina Hiseq 2000 platform and 100-base paired-end reads were generated.

### Sequencing data analysis

Raw data (raw reads) of fastq format were first processed through in-house PERL scripts. In this step, clean data (clean reads) were obtained by removing reads containing adapter, reads containing ploy-N and low quality reads from raw data. At the same time, Q20, Q30 and GC content of the clean data were calculated. All the downstream analyses were based on the clean data with high quality. Reference genome and gene model annotation files were downloaded from genome website directly (ftp://ftp.ensemblgenomes.org/pub/release-23/plants/fasta/vitis_vinifera/ dna/). Index of the reference genome was built using Bowtie v2.0.6 and base paired-end clean reads were aligned to the reference genome using TopHat v2.0.9. We selected TopHat as the mapping tool since TopHat can generate a database of splice junctions based on the gene model annotation file and thus a better mapping result than other non-splice mapping tools.

HTSeq v0.6.1 was used to count the reads numbers mapped to each gene. And then RPKM of each gene was calculated based on the length of the gene and reads count mapped to this gene. RPKM (Reads Per Kilobase of exon model per Million mapped reads) was used for the effect of sequencing depth and gene length for the reads count at the same time, and is currently the most commonly used method for estimating gene expression levels [96].

### Differential expression analysis

Differential expression analysis of two conditions/groups (two biological replicates per condition) was performed using the DESeq R package (1.10.1). DESeq provided statistical routines for determining differential expression in digital gene expression data using a model based on the negative binomial distribution. The resulting P-values were adjusted using the Benjamini and Hochberg’s approach for controlling the false discovery rate. Genes with an adjusted *P-value* < 0.05 selected by DESeq were assigned as differentially expressed.

### Selection of candidate genes

We used Gene Ontology (GO) and KEGG enrichment analysis of differentially expressed genes to select candidate genes. GO enrichment analysis of differentially expressed genes was implemented by the GOseq R package, in which gene length bias was corrected. GO terms with corrected P-value less than 0.05 were considered significantly enriched by differential expressed genes. KEGG is a database resource for understanding high-level functions and utilities of the biological system, such as the cell, the organism and the ecosystem, from molecular-level information, especially large-scale molecular datasets generated by genome sequencing and other high-through put experimental technologies (http://www.genome.jp/kegg/). We used KOBAS software to test the statistical enrichment of differential expression genes in KEGG pathways. We also used the differentially expressed genes clustering methodology to find the candidate genes. The clustering analysis was made based on the expression pattern of the differentially expressed genes. We selected the genes that the expression patterns were very similar to the genes closely related to the anthocyanins biosynthesis as the candidate genes.

### qRT-PCR analysis

Thirty genes were chosen for validation using qRT-PCR. Specific primer pairs for selected genes used in qRT-PCR were designed as shown in Additional file 3: Table S3. The cDNA was transcribed from 1 μg of total RNA using the TOYOBO ReverTra Ace qPCR RT Master Mix (TOYOBO, Japan) in 10 μL of reaction mixture. The qRT-PCR was performed with the Roche LightCycler480 Real-Time Detection System (Roche) with Roche SYBR Green I (Roche, USA). The thermal profile for SYBR Green I RT-PCR was 95 °C for 15 min, followed by 40 cycles of 95 °C for 10s and 55 °C for 30s and 72 °C for 30s. Each plate was repeated three times in independent runs for all reference and selected genes. The reference gene (Actin) was used for normalization. The comparative CT method (2^-ΔΔCT^ method) was used to analyze the expression levels of the different genes [97].

### SNPs of candidate gene analysis

Picard-tools v1.96 and samtools v0.1.18 were used to sort, mark duplicated reads and reorder the bam alignment results of each sample. GATK2 software was used to perform SNP calling.

### Statistical analysis

All of the experiments analyzed using data comparisons were repeated three times. Statistical analyses were performed using variance (ANOVA) followed by Duncan’s new multiple range tests with SPSS version 17.0 (SPSS, Chicago, IL, USA). A significance level of p < 0.01 was applied.

## Additional files

Additional file 1: Table S1. B2vsW2.DEG_GO_enrichment_result. (XLS 147 kb)
Additional file 2: Table S2. Differential expression *UGT*s between W3 and W1. (XLS 29 kb)
Additional file 3: Table S3. Primers for qRT-PCR. (XLS 25 kb)

## Abbreviations

4CL: 4-coumaroyl-coA synthase
5GT: UDP-glucose:anthocyanin-5-*O*-glucosyltransferase
AAT: Anthocyanin acyltransferases
ABC: ATP binding cassette
AM: anthoMATEs
ANR: Anthocyanidin reductase
bHLH: Basic helix–loop-helix proteins
bp: Base pair
C4H: Cinnamate-4-hydroxylase
cDNA: Complementary DNA
CHI: Chalcone isomerase
CHS: Chalcone synthase
coum: Coumaryl
cv: Cultivar
Cy: Cyanidin
DAF: Day after flowering
DEG: Differential expression gene
DFR: Dihydroflavonol 4-reductase
diglu: Diglucoside
Dp: Delphinidin
ESI: Electrospray ionization
ESI: Electrospray ionization
F3′5′H: Flavonoid-3′5′-hydroxylase
F3′H: Flavonoid-3′-hydroxylase
F3H: Flavanone-3β-hydroxylase
FLS: Flavonol synthase
glu: Glucoside
GO: Gene Ontology
GST: Glutathione S-transferase
LAR: Leucoanthocyanidin reductase
LDOX: Leucoanthocyanidin dioxygenase
MATE: Toxic compound extrusion
MRM: Multiple reaction monitoring
MRM: Multiple reaction monitoring
MRP: Multidrug resistance-associated protein
Mv: Malvidin
OMT: *O-*methyltransferase
PAL: Phenylalanine ammonia lyase
Pg: Pelargonidin
Pn: Peonidin
Pt: Petunidin
qRT-PCR: Quantitative real time poly-merase chain reaction
QTL: Quantitative trait locus
RPKM: Reads Per Kilobase of exon model per Million mapped reads
SAM: S-adenosyl-L-methionine
SCPL: Serine carboxypeptidase-like
SNP: Single nucleotide polymorphism
UFGT: UDP-glucose:flavonoid-3-*O*-glucosyltransferase
UPLC: Ultra Performance Liquid Chromatography
